# Outcomes and toxicity of oral Fosfestrol in metastatic castration-resistant prostate cancer—a real-world experience

**DOI:** 10.3332/ecancer.2023.1589

**Published:** 2023-08-14

**Authors:** R Nandini Devi, VP Praveen Kumar Shenoy, Irshad Ismail, Manuprasad Avaronnan

**Affiliations:** Department of Clinical Hematology and Medical Oncology, Malabar Cancer Centre, Thalassery, Kannur 670103, India

**Keywords:** castration-resistant prostate cancer, Fosfestrol, hormonal therapy, LMIC

## Abstract

**Introduction:**

Although there are multiple drugs approved for the treatment of metastatic castration-resistant prostate cancer (CRPC), the cost can be a limiting factor in using them in a resource-limited setting. Therefore, less expensive alternatives are the need of the hour. We have been using Fosfestrol which is a cheap and orally administered oestrogen analogue in metastatic CRPC. We carried out a retrospective study to analyse its efficacy and toxicity.

**Results:**

A total of 65 patients received Fosfestrol during 2015–2020. The median age was 65 years (range 50–83 years). Thirty-four patients (53%) had other medical comorbidities. Skeletal-only metastasis was the commonest pattern of metastasis (*n* = 41, 64%) followed by skeletal with nodal metastasis (*n* = 15, 23%). The majority of the patients had undergone upfront surgical castration (*n* = 60, 93%). All the patients had adenocarcinoma and 38 patients (58%) had a high Gleason’s score. Forty-one patients (63%) had a prostate-specific antigen (PSA) response (decrease of ≥50% in the PSA concentration from the pre-treatment baseline PSA value) and 54 patients (83%) had a symptomatic response. At the end of a median follow-up of 16 months, the median progression-free survival (PFS) was 8.3 months (CI 4.7–11.8) and the median overall survival (OS) was 27.5 months (CI 25.4–29.5). PSA response and prior treatment with abiraterone acetate were found to have a significant association with survival outcomes. Patients with PSA response had better median PFS and OS; while patients who have received prior abiraterone acetate therapy had worse survival outcomes. Twenty-nine patients (45%) received some form of subsequent treatment after stopping Fosfestrol. The most common oxicity observed was thrombosis (*n* = 9, 13%) followed by gynecomastia (*n* = 4, 6%).

**Conclusion:**

We conclude that oral Fosfestrol is a cheap and effective agent in the armamentarium against metastatic CRPC and warrants further studies in a clinical trial setting.

## Background

Carcinoma prostate is the second most common cancer in males with around 1,414,259 (7.3%) new cases and 375,304 (3.8%) deaths annually [1]. As the majority of patients with prostate cancer present in the early stage, 5-year survival rates are as high as 97% in developed countries [2]. But all patients who present with advanced disease progress to the castration-resistant stage, treatment of which is challenging. The therapeutic landscape of castration-resistant prostate cancer (CRPC) has dramatically changed over the last few years with the approval of many novel agents including androgen receptor antagonists like enzalutamide or darolutamide, immunotherapy like Sipuleucel-t or Pembrolizumab, and targeted agents like poly(ADP-ribose) polymerase (PARP) inhibitors [3]. But the exact sequencing of these agents to achieve maximum survival rates remains debatable. Also, most of these drugs are expensive and the majority of patients in developing countries do not have access to them [4].

As prostate cancer is an androgen-dependent cancer, androgen deprivation therapy (ADT) was the standard first-line therapy until the role of Docetaxel in hormone naïve metastatic prostate cancer was established in 2014 [5]. Second-line hormonal agents approved in CRPC also act on the androgen synthesis pathways or receptors [6, 7]. Another hormonal strategy found to be effective in prostate cancer is the use of exogenous oestrogens. Exogenous oestrogens may exert negative feedback inhibition on the pituitary gland with a resultant reduction in luteinising hormone and subsequent fall in testosterone levels. But later on, these agents fell out of favour due to adverse events related to high-dose oestrogens, lack of proven survival benefit and the emergence of newer forms of hormonal therapies. Diethylstilbestrol (DES) and its analogues were the most common oestrogen derivatives that were studied. DES increases the level of sex hormone binding globulin which also reduces the free testosterone levels. DES is also known to have a direct cytotoxic effect on prostatic cancer cells by transforming growth factor beta (TGF-β) upregulation and apoptosis [8]. But cardiovascular and thrombotic events were a major concern in many of the studies using DES [9].

Fosfestrol tetrasodium – DES diphosphate – is a synthetic oestrogen derivative which is available in parenteral as well as oral formulations [10]. Oral Fosfestrol is advantageous compared to DES, due to the following reasons (a) Fosfestrol is a non-toxic prodrug of DES (b) It can be administered safely/easily (c) It selectively accumulates in prostatic cancer cells and (d) exerts its cytotoxic effect only after conversion to active metabolite DES. These active metabolites are said to inhibit the electron flow from Ubiquinone to Cytochrome C1 in mitochondria [11, 12]. There are only very few studies examining the efficacy of oral Fosfestrol. In a study of 38 patients with CRPC, Orlando *et al* [13] reported an improvement in pain score in 53% of patients and a median progression-free survival (PFS) of 7 months. This study, which used low-dose Fosfestrol (100 mg three times a day), also reported significant toxicities including peripheral oedema, worsening of gynaecomastia and deep vein thrombosis. Another study from India reported the use of oral Fosfestrol (120 mg three times a day) in 47 patients with CRPC. In this study, 55% of the patients had PSA response >50% and had a median survival of 14 months after initiation of Fosfestrol. But unlike the initial study thrombotic complications were not observed in this group and the most common side effect was gastrointestinal side effects (6%) [14]. In order to circumvent the first pass hepatic metabolism of oestrogen analogues and thereby prevent thrombotic complications, transdermal oestrogen patches are being studied in the management of CRPC in the UK PATCH study and results are awaited. Once available the transdermal oestrogen patch might be a patient-friendly and safer therapeutic approach [15]. In a low-middle-income country like India, where the newer agents cannot be afforded by a majority of patients, Fosfestrol can be an important drug. Also, as the optimal sequencing of all available agents is not yet defined, Fosfestrol may have its own place in the armamentarium against CRPC. Here we report our experience with oral Fosfestrol in metastatic CRPC.

## Materials and methods

This is a retrospective study conducted at the Medical Oncology Department of a tertiary referral oncology centre in North Kerala. The study was approved by the Institution Review Board. The primary objective of the study was to find out the response rates, PFS, and toxicities with Fosfestrol in CRPC. The secondary objective was to estimate the overall survival (OS) with Fosfestrol. All patients with CRPC who received Fosfestrol at least for 1 month, during the period of 2015–2020, were included. Patients with incomplete data and patients initiated on Fosfestrol from outside were excluded. Details about baseline characteristics, prior treatment, toxicities observed and outcomes of Fosfestrol therapy were collected from the case records. The following operational definitions were used:

1. CRPC was defined as two consecutive increases in the PSA concentration (over a reference value) or radiographic evidence of disease progression with a serum testosterone level of 50 ng per decilitre or less (≤2.0 nmol per litre) in patients with metastatic prostate cancer. Patients who underwent bilateral orchidectomy were included even if serum testosterone levels were unavailable.
2. PSA response was defined as a decrease of ≥50% in the PSA concentration from the pre-treatment PSA value, which was confirmed after ≥4 weeks by one more value.
3. PFS was calculated from the date of the start of Fosfestrol to the date of progression. Progression is considered when there is a progression in at least 2 of 3 domains – biochemical, radiological or symptomatic progression.
4. OS was calculated from the date of the start of Fosfestrol till the date of the last follow-up or death
5. Biochemical progression was defined as a serial rise in S.PSA on 3 consecutive tests at least 4 weeks apart

All the patients were started on oral Fosfestrol at a dose of 120 mg three times a day. They underwent 1–3 monthly clinical examinations and S.PSA estimations. All patients with biochemical progression or symptomatic progression undergo radiologic examination. Fosfestrol was continued till progression or till grade 3/4 toxicity.

### Statistical methods

Being a retrospective study, sample size calculation was not performed for this study. Statistical analysis was done using the Statistical Package for the Social Sciences version 20 (IBM Corp., Armonk, NY, USA). Descriptive analysis was used for frequency and percentages. Prognostic factors for survival were identified by univariate analysis using the log-rank test. Also, prognostic factors were tested using the Cox regression for the multivariate analysis. *p*-value < 0.05 was considered statistically significant.

## Results

### Baseline characteristics

A total of 65 patients received Fosfestrol during the study period. The median age was 65 years (50–83 years). The baseline Eastern Cooperative Oncology Group (ECOG) performance status was PS-1 in 7 (10.8%) patients, PS-2 in 44 (67.7%) and PS-3 in 5(7.7%) patients. Thirty-four patients (53%) had other medical comorbidities. The most common co-morbidity was systemic hypertension in 15 (23.1%) patients and type 2 diabetes mellitus in 11 (16.9%) patients. All the patients had prostatic adenocarcinoma. Skeletal-only metastasis was the commonest pattern of metastasis (*n* = 41, 64%) followed by skeletal with nodal metastasis (*n* = 15, 23%). The majority of the patients had upfront surgical castration (*n* = 60, 93%). The majority (*n* = 50, 76.9%) had Gleason’s score (GS) more than 6. Fifteen patients had received prior Abiraterone, 26 patients received prior Docetaxel and 8 patients received both Docetaxel and Abiraterone. Other baseline characteristics are shown in Table 1.

### Outcomes

**Figure 1.**
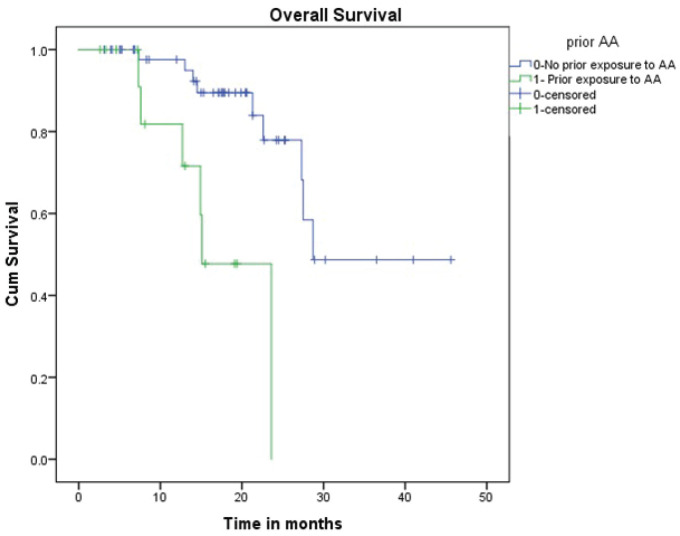
OS based on prior abiraterone acetate therapy.

**Figure 2.**
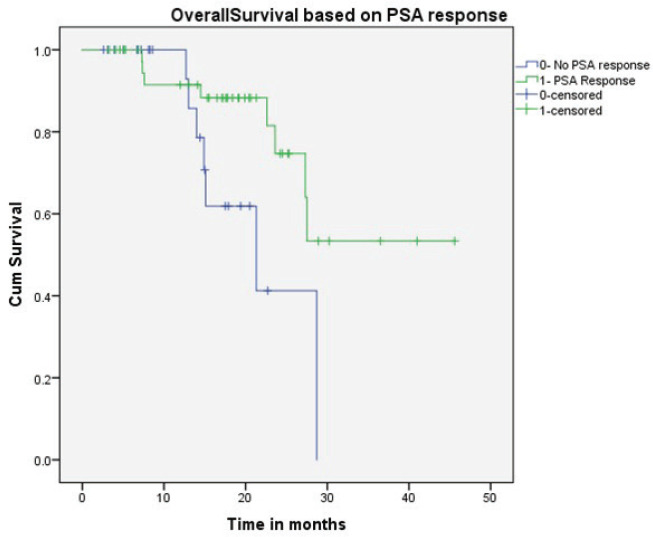
OS based on PSA response.

All patients received oral Fosfestrol at the dose of 120 mg thrice daily. Fifty-four patients (83%) had a symptomatic response. Forty-one patients (63%) had a PSA response, of which 13 (20%) patients had normalisation of PSA (complete response). At the end of a median follow-up of 16 months, median PFS was 8.3 months (CI 4.7–11.8) and the median OS was 27.5 months (CI 25.4–29.5). On univariate analysis, PSA response and prior exposure to abiraterone acetate were predictive of survival. The median OS was significantly longer for patients who received Fosfestrol prior to abiraterone acetate (28.7 versus 15 months, *p*-value < 0.001). Also, patients who achieved PSA response had better OS compared to those who did not (NR versus 21 months, *p*-value 0.027). The use of Fosfestrol prior to abiraterone acetate and PSA response were found to be predictors of longer survival in the multivariate analysis also (Figures 1 and 2). The PFS was better in patients with PSA response (12 versus 5 months, *p*-value 0.001). In patients who received Fosfestrol prior to abiraterone, PFS was numerically longer but there was no statistically significant difference (11 versus 7 months, *p*-value 0.060). The ECOG performance status at the time of initiation of Fosfestrol was associated with better PFS in univariate analysis, but there was no difference in OS. Also, there was no statistically significant difference in OS or PFS according to prior use of docetaxel, and the site of metastasis. The univariate analysis results are summarised in Table 2. Twenty-nine patients received some form of subsequent treatment. The subsequent therapies were abiraterone acetate in 25 patients, Docetaxel in 4 patients and enzalutamide in 3 patients. One patient each received Cabazitaxel and Ketoconazole.

### Toxicity

Sixteen (24%) patients had adverse reactions, and in three patients, it led to treatment discontinuation. Deep vein thrombosis was seen in 9 (13.8%) patients and gynecomastia in 4 (6%) patients. All the cases of deep vein thrombosis involved the lower limbs. None of our patients had pulmonary thromboembolism. The cause for treatment discontinuation was disease progression in 46 patients, thrombosis in 3 patients and not available in rest. No deaths occurred due to thrombotic complications.

## Discussion

In our study of 65 patients, oral Fosfestrol showed favourable outcomes in metastatic CRPC with a manageable toxicity profile. The majority of our patients underwent surgical castration as upfront ADT and had bone metastasis. About half of the patients received Fosfestrol as the first-line treatment for CRPC. More than 80% of our patients had a symptomatic response and 60% had a PSA response. Median PFS was 8.3 months and OS was 27.5 months. Survival was better in patients who did not have prior abiraterone exposure and those who had a PSA response. Only a minority of our patients had significant toxicities and thrombotic events were an important adverse event. Our study showed that this cheap and widely available oral drug can be an important agent in the management of CRPC, especially in a resource-limited setting.

Orlando *et al* [13] reported an overall response rate of 79% and symptom response of 53% with low-dose oral Fosfestrol. Siddiqui *et al* [16] described a 50% PSA response with a median response duration of 12.7 months. In an Indian study, oral Fosfestrol demonstrated symptomatic response in 61% and PSA response in 55% of patients [14]. In our study, 83% of patients had symptomatic response and 63% of patients had PSA response, both of which are superior compared to the aforementioned studies. One reason for the better response rates in our study may be related to its use in earlier lines of treatment. Also, the response rates with Fosfestrol are comparable to other agents approved in CRPC like abiraterone acetate, enzalutamide, and taxane chemotherapy [6, 17–19]. Table 3 shows the comparison of our study with others.

In our study, patients with PSA response had better OS as well as PFS, which was consistent with previous literature. The median OS described by Orlando *et al* [13] was 13 months in PSA responders compared to 7 months in non-responders. Similarly, Kalaiyarasi *et al* [14] reported a median OS of 20.8 months in responders (versus 4.8 m in non-responders). An interesting observation was the negative impact of prior abiraterone exposure on survival. Patients who had received abiraterone prior had inferior OS of 15.1 months (versus 28.7 m in Abiraterone naive) which means prior abiraterone therapy might be conferring cross resistance to Fosfestrol therapy. There is ample retrospective data and a prospective trial proving cross-resistance between abiraterone and enzalutamide [20]. We think similar mechanisms of cross-resistance might be responsible for the inferior survival in our study post abiraterone therapy. Hence, we think that Fosfestrol at earlier lines might be an efficient and cost-effective therapeutic choice for CRPC. Omlin *et al* [21] have demonstrated a favourable PSA response with abiraterone therapy in DES-treated patients. This is in keeping with our observation that Fosfestrol might be beneficial at an earlier line and any further disease progression can be effectively salvaged with other hormonal agents like abiraterone. The cross-resistance between various hormonal therapies needs to be explored further as this is important for proper sequencing of therapies in CRPC. Although ECOG performance status was found to be associated with PFS, the number of patients with a performance status of one or three was too small to draw any definite conclusions.

The toxicities observed were slightly different among all these studies. The most commonly reported adverse effect was gynaecomastia and gastrointestinal disturbances, including diarrhoea and transaminitis. The incidence of thrombotic complications varied the most. In the study by Siddiqui *et al* [16], no thrombotic events were observed. But in this study, anti-thrombotic prophylaxis with warfarin and aspirin was given, which might have prevented the thrombotic events. Kalaiyarasi *et al* [14] also reported no thrombotic complications and they had suggested genetic variation between Caucasian and Indian people as a possible explanation for the lesser thrombotic complications. In our study, around 9 (13.8%) patients had deep vein thrombosis and in 3 patients it led to treatment discontinuation confirming the thrombotic risk of oestrogens in our population. But it cannot be ruled out if the thrombotic risk is mainly related to the prostatic malignancy or other factors like co-morbidities.

Our study is the largest report proving the efficacy of oral Fosfestrol in CRPC. The use of Fosfestrol in the earlier lines of treatment clearly showed impressive response rates and survival comparable to other novel agents. Also, it was noted that using Fosfestrol prior to abiraterone has led to improved survival which can be important information in the sequencing of drugs in CRPC, especially in a resource-limited setting. The pharmacoeconomic implications of Fosfestrol are particularly appealing. Fosfestrol is a cost-effective choice among the hormonal therapies of CRPC. The cost for abiraterone acetate/Prednisolone is 28,800 INR/year, for Fosfestrol is 27,600 INR/year and for enzalutamide 1.92 lakhs INR/year. The median PFS for Fosfestrol in our study was 12.3 months, so when comparing Fosfestrol versus abiraterone, an additional 1,230 INR will be saved per person per year. This is particularly important when these therapies are partly/fully supported by government funds. Not to mention additional costs involved in the monitoring of dyselectrolytemia and hyperglycaemia and their subsequent treatment while on abiraterone therapy [22, 23]. Although thrombotic events are reported in the study, many of the patients with multiple comorbidities including coronary artery disease tolerated the drug well. The limitation of our study includes a small sample size and retrospective design leading to inadequate capture of minor adverse events. Though clinical and biochemical response was assessed regularly, the radiological assessment was done only when clinically indicated.

## Conclusion

Our study shows that Fosfestrol is an effective drug in metastatic CRPC with a manageable toxicity profile and it has an important role in the sequencing of drugs in CRPC. In view of the low cost and wide availability, it must be studied in a randomised clinical trial setting to confirm its efficacy.

## Conflicts of interest

The authors declare that they have no conflict of interest.

## Funding

There were no external sources of funding for this project.

## Figures and Tables

**Table 1.** Baseline characteristics.

Characteristics	*N* (%)
Age (years) ≤50 50–70 70–80 ≥ 80	1 (1.5%) 49 (75.3%) 13 (20%) 2 (3%)
GS Low (≤6) Intermediate (7) High (8–10) Not available	5 (7.6%) 12 (18.4%) 38 (58.4) 10 (15.3%)
Sites of metastases Bone only Lymph nodes Visceral	41 (63%) 2 (3.1%) 2 (3.1 %)
Line of treatment First line Second line Third line	32 (49.2%) 25 (38.4%) 8 (12.3%)
Prior therapies Upfront ADT (surgical versus medical) Prior docetaxel Prior abiraterone acetate Both	60 (93%) versus 5 (7.6%) 26 (40%) 15 (23%) 8 (12.3%)
PSA at start of Fosfestrol Less than/equal to 20 More than 20	19 (29.2%) 46 (70.76%)

**Table 2.** Univariate analysis of PFS and OS.

Factors	PFS (months)	*p*-value	OS (months)	*p*-value
ECOG PS (1 versus 2 versus 3)	25.9 versus 7.5 versus 4.9	0.015	27 versus NR versus 13	0.416
Site of metastases (Bone only versus Nodal/visceral)	7.7 versus 9.1	0.690	28.7 versus 27.3	0.570
Prior docetaxel (yes versus no)	7.4 versus 8.2	0.459	27.5 versus NR	0.622
Prior abiraterone acetate (yes versus no)	11 versus 7	0.060	28.7 versus 15	<0.001
PSA response	12 versus 5	0.001	NR versus 21	0.027

**Table 3.** Comparison of our study with similar studies.

	Orlando *et al* [13]	Siddiqui *et al* [16]	Kalaiyarasi *et al* [14]	Our study
Study period	1992–1998	1991–2001	2012–2015	2015–2020
No of patients	38	12	47	65
Median age in years (range)	70 (58–89)	66 (57–73)	67 (46–85)	65 (50–83)
Prior treatment lines median (range)	3 (2–6)	NA	2 (2–4)	2(1–3)
Treatment schedule	100 mg 8 hourly	500 mg IV infusion day 1 f/b 1,000 mg IV infusion day 2–6 f/b Oral Fosfestrol 120 mg 8 hourly daily	120 mg PO 8 hourly	120 mg PO 8 hourly
Symptom response (%)	53	NA	61	83
PSA response (%)	58	50	55	63
Complete response rate (%)	21	NA	28	20
Mean response duration (months)	3.5	12.7	NA	13.1
Median PFS (months)	7	3.9	6.8	8.3
Median OS (months)	12	NA	14.7	27.5
Median OS PSA responders Non-responders (months)	13 7	NA	20.8 4.8	NR (*p* 0.027) 21
Median PFS PSA responders Non-responders (months)	NA	NA	12.3 1.8	12 (*p* 0.001) 5
Adverse reactions (%)	Gynecomastia 38 Oedema 32 GI discomfort 19 DVT 8 Skin rash 5 Hypertension 5 Transaminitis 2	Gynaecomastia 8 CHF 8	GI ‑ 6 Transaminitis‑ 2	DVT 9 Gynaecomastia 6
